# Advancing precision in histocompatibility and immunogenetics: a comprehensive review of the UCLA exchange program

**DOI:** 10.3389/fgene.2024.1352764

**Published:** 2024-02-01

**Authors:** Qiuheng Zhang, Arlene F. Locke, Andrea Carolina Alvarez, Maria L. Cabarong, Lek Ching Liv, Belen Garcia P. Alfaro, David W. Gjertson, Elaine F. Reed

**Affiliations:** UCLA Immunogenetics Center, Department of Pathology and Laboratory Medicine, David Geffen School of Medicine, University of California, Los Angeles, Los Angeles, CA, United States

**Keywords:** HLA, KIR, MICA, virtual crossmatch, physical crossmatch, HLA antibody

## Abstract

Precise typing of human leukocyte antigens (HLA) is crucial for clinical hematopoietic stem cell and solid organ transplantations, transfusion medicine, HLA-related disease association, and drug hypersensitivity analysis. The UCLA Cell Exchange program has played a vital role in providing educational and proficiency testing surveys to HLA laboratories worldwide for the past 5 decades. This article highlights the significant contribution of the UCLA Cell and DNA Exchange Programs in advancing HLA antibody testing, genotyping, crossmatches, and, more recently, virtual crossmatches. Additionally, we discuss future directions of the UCLA Cell Exchange program to support histocompatibility testing to adapt to the fast-evolving field of immunotherapy, tolerance and xenotransplantation.

## Introduction

The UCLA HLA Clinical Laboratory Exchange Program has played a pivotal role in advancing the field of Human Leukocyte Antigen (HLA) testing and transplantation diagnostics for nearly 50 years. Established in 1974 with a vision to foster international collaboration, to exchange knowledge, and to advance transplant immunology research, the program has evolved over the years from one that initially provided challenges just for serological-based tests to one that now sends analytes appropriate for molecular-based assays. Many of the cell lines cultured and sent out by the program have been used as reference cells in the International Histocompatibility Workshops. In 1993, the original aim of the program of internal laboratory quality control and standardization of HLA antigen-level typing reagents was modified to incorporate proficiency testing (PT) for accreditation of HLA allele-level typing through the HLA DNA exchange. Subsequently, we now provide PT for several of our surveys including HLA serum antibody identification, cytotoxicity and flow cytometry crossmatch tests, and Killer-cell Immunoglobulin-like Receptors (KIR) genes. Online reporting was added in 2011, in which labs submit results though a website: https://cell-exch.ctrl.ucla.edu/register/. Exchange results are sent to all participating centers, summarized at ASHI annual meetings, and periodically published as milestone reports (Loon et al., 1987; Lau et al., 1989; Lau et al., 1990; Park et al., 1994; Locke et al., 2023). Participants of UCLA Exchange Program include laboratories from 30 countries worldwide. In this article, we present a fresh account of the impact of the UCLA HLA Clinical Laboratory Exchange Program in the field of Histocompatibility and Immunogenetics.

### HLA typing

**FIGURE 1:**
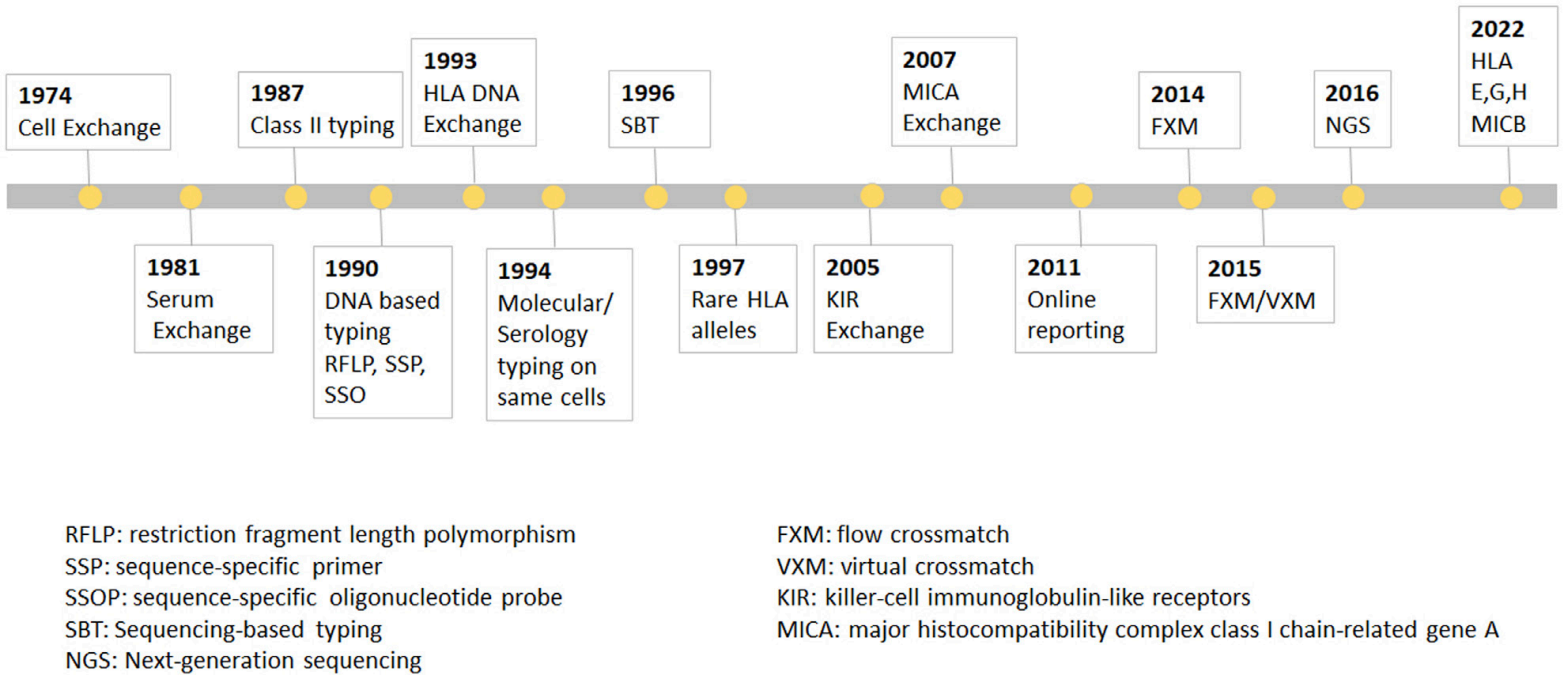
The UCLA cell Exchange Program. The UCLA cell Exchange Program was first established in 1974 followed by serum exchange in 1981. In 1987, HLA class II typing was initiated with first shipment of two lymphoblastoid cell lines. By 1990, DNA typing, including restriction fragment length polymorphism (RFLP), PCR-sequence-specific primer (PCR-SSP) and PCR-sequence-specific oligonucleotide probe (PCR-SSOP) was implemented in the HLA DNA exchange survey, which became the first graded proficiency test service offered by the UCLA Exchange program in 1993. Subsequently, PCR-sequencing-based typing (PCR-SBT) was added 1996. In 2016, the exchange program embraced cutting-edge methodologies for the next-generation sequencing (NGS) HLA tying.

Since the first discovery of the HLA-A2 antigen in 1958, the field of Immunogenetics and Histocompatibility has seen tremendous advancement (Dausset, 1958). Today, the field plays important roles in disease association, transfusion support, solid organ transplantation (Bosanquet et al., 2015; Wehmeier et al., 2017; Zhang et al., 2018; Frischknecht et al., 2022) and hematopoietic stem cell transplantation (HCT) (Morishima et al., 2015). Advancements were slow at first due the extreme polymorphic and multi-locus nature of the HLA system, and the lack of standardization among serological typing reagents. Establishing reproducible relationships between antigens and functional polymorphisms by a single laboratory was problematic. Consequently, International Histocompatibility Workshops were organized by WHO bringing together a handful of established laboratories (designated as “reference” labs) who exchanged their reagents, methodologies and results with all participating workshop laboratories with the goal of standardizing results. The UCLA International Cell Exchange was launched in 1974 to continue this collaboration with 85 participating laboratories, which then expanded to more than 290 participates worldwide by 1997 (Figure 1). During its early years, the UCLA exchange program focused on building relationships with international HLA laboratories. These exchanges led to significant breakthroughs, enhancing the accuracy and efficiency of HLA typing (Lau et al., 1989; Lau et al., 1990). For example, the percent agreement in detection of the antigen HLA-A23 among laboratories went from 30% to 97% over the 23-year period (1974–1997).

One of the major challenges of serological HLA typing was the need of viable cells. The UCLA Cell Exchange was made possible with the breakthrough of shipping viable lymphocytes at room temperature worldwide and later on provide a reliable shipping method (Park and Terasaki, 1974). As efforts were made to standardize specificities for international consensus, the exchanges identified instances of duplicate names for the same specificity and identical names assigned to different specificities. Examples of variants which were extensively studied in previous cell exchanges and received formal designations by the WHO Nomenclature Committee are: A9.3 (A*24:03), BN21 (B*40:05), B5.35 (B*51:02), 5Y/8w58/BSNA (B*78:01, B*78:02), numerous B15 variants (B*15:08, B*15:11, B*15:12, B*15:15), and DT (B*81:01). The Cell Exchange data has provided vital correlation between alleles and serologic names in many cases, such as establishing B*15:18 as B71 and Cw*17:01 as a short Cw7.

In 1987, HLA class II typing was initiated with first shipment of two lymphoblastoid cell lines. By 1990, DNA typing, including restriction fragment length polymorphism (RFLP), PCR-sequence-specific primer (PCR-SSP) and PCR-sequence-specific oligonucleotide probe (PCR-SSOP) was implemented in the HLA DNA exchange survey, which, as was mentioned above, became the first graded proficiency test service offered by the UCLA Exchange program in 1993. Subsequently, PCR-sequencing-based typing (PCR-SBT) was added 1996. In 2016, the exchange program embraced cutting-edge methodologies for the next-generation sequencing (NGS) HLA typing. The Cell Exchange has also been graded since 2018 and many laboratories have used the exchange to satisfy their proficiency test requirement for clinical laboratory accreditation. In 1994, the Cell Exchange initiated offering the same cells for molecular typing as for serologic typing for Class I. The data from the parallel typing was instrumental in identifying serologic equivalents for Class I and Class II alleles that previously had little or no serologic information.

In contradistinction to other histocompatibility PT programs (e.g., ASHI and CAP), the UCLA International Cell Exchange has often focused on uncommon HLA alleles. This provides laboratories and companies to validate and improve their HLA typing techniques and reagents (Supplementary Table S1). Since 1994, a total of 120 alleles (21 HLA-A locus, 49 -B locus, 7 -C locus, 1 DQB1, 38 DRB1 and 4 DRB3/4/5) typed in exchange cells were considered uncommon, as listed in Supplementary Table S1. Often, these challenging HLA types were found to include “variants,” which represented new alleles that were not defined by serology. In addition, certain HLA alleles do not possess a serologically defined antigenic counterpart. As a result, it is not consistently feasible to associate a serological equivalent with each HLA allele. This information was routinely added to the HLA Dictionary. The data from the UCLA Cell Exchange has been invaluable in establishing correlations between alleles and serologic names. From 1974 to 2021, a total of 1704 cells were sent out worldwide. Among them, 14 cells were initially typed in the Cell Exchange and now serve as reference cells. The UCLA cell exchange has greatly contributed to the HLA Dictionaries (Schreuder et al., 1999; Schreuder et al., 2001; Holdsworth et al., 2009), as well the publication of Common and Well documented alleles (Cano et al., 2007).

### HLA antibody detection and identification

Donor-specific alloantibody (DSA) either present at the time of transplantation or arising *de novo* posttransplant is a risk factor for antibody mediated rejection (AMR) and potentially allograft loss in solid organ transplants (Lefaucheur et al., 2023). The development of HLA antibody detection has been significantly advanced in clinical transplantation over the past decades. The initial approach for identifying anti-HLA antibodies involved the use of the complement‐dependent cytotoxicity (CDC) assay, a method pioneered by Terasaki and McClelland in 1964 (Terasaki and McClelland, 1964). Over the past 50 years, prospective CDC crossmatches and flow crossmatches (FXM) have been standard practices for solid organ transplantation to detect donor-specific reactivity. For CDC crossmatch, donor lymphocytes and recipient serum are mixed with complement. The membrane attack complex forms when DSA bind to donor HLA antigens on the cell surface leading to donor cell lysis. The CDC assay is a functional test, but with a low sensitivity that only detects high titered complement fixing antibodies. The FXM introduced in early 80s significantly increased the sensitivity of the lymphocyte crossmatch test (Garovoy et al., 1983). In the mid-90s, the introduction of HLA antibody detection by flow cytometry and Luminex technology using purified HLA class I and II antigens on solid phase platforms have revolutionized the ability to detect HLA antibodies with high sensitivity and specificity. However, solid phase assays are also subject to issues with prozone (Schnaidt et al., 2011), interfering substances (Goldsmith et al., 2020) and false positive reactivities to cryptic epitopes (El-Awar et al., 2009). UCLA Serum Exchange was initiated in 1981 for HLA antibody identification. Since then, more than 1,300 well characterized reference sera have been sent out to participating laboratories for HLA antibody evaluation. The goal is to facilitate HLA laboratory to accurately detect the presence of HLA antibodies, HLA antibody specificity identification and crossmatching. For single HLA class I and class II antigen bead (SAB) testing, data collected include serum pre-treatment, median fluorescence intensity (MFI) cut-off, vendor, and reagent lot numbers. The UCLA Serum Exchange provides the concordance and discordance in HLA antibody detection across multiple laboratories as well as intra- and inter laboratory variability to participating laboratories. The exchange results recently showed that laboratories using sera pretreated with DTT or EDTA have 10%–15% less variability compared to laboratories not using serum pre-treatment (Locke et al., 2023).

**FIGURE 2:**
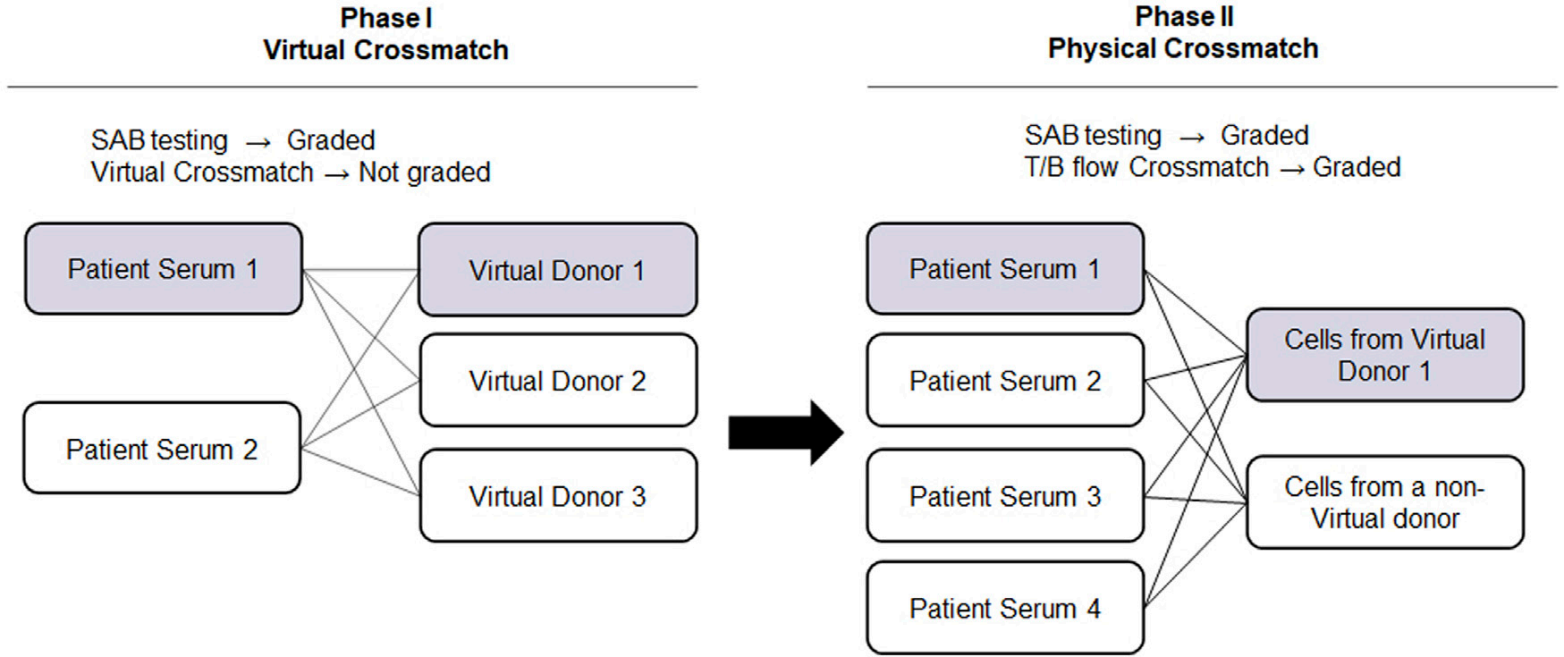
UCLA Virtual Crossmatch Exchange Survey. UCLA VXM Exchange consist of two Phases. In Phase I, two serum samples and three virtual donors are provided to the laboratories to perform SAB tests and 6 VXM. In Phase II, four recipient serum samples and lymphocytes from two donors are provided to perform SAB tests and 8 FXM. In Phase II, one recipient-donor pair will be selected from the VXM of Phase I, therefore a direct comparison between FXM and VXM can be achieved. VXM, virtual crossmatch; SAB, single antigen bead test; FXM, flow cytometry crossmatch; MFI, median fluorescence intensity.

The advancement of solid phase assays, particularly the SAB assay, allows laboratories to predict physical crossmatch (PXM) results with high accuracy. The American Society for Histocompatibility and Immunogenetics defines virtual crossmatch (VXM) as an assessment of immunologic compatibility based on patient’s alloantibody profile compared with donor’s histocompatibility antigens. However, the VXM is performed based on the agreement between the transplant centers with their supporting HLA laboratories and it is highly variable from center to center. To address this gap, in 2015, UCLA Virtual Crossmatch Exchange was launched. It is a two-phase challenge that assesses laboratory consensus in HLA antibody detection, VXM and FXM reporting. The UCLA VXM Exchange is the first program designed to provide laboratories with the opportunity to compare VXM with an actual FXM. In Phase I, participating laboratories are sent two sera for HLA Class I and Class II antibody testing by SAB and VXM with the complete HLA typing of 3 virtual donors (HLA A, B, C, DRB1/3/4/5, DQA1, DQB1, DPA1, and DPB1) for a total of six VXM challenges. Each VXM challenge is given a fictional clinical vignette including if the patient is a primary or regraft recipient based on prior transplantation. In Phase II, laboratories that are part of the program receive four recipient sera samples and lymphocytes from two donors. These samples are used for SAB testing and FXM. An interesting aspect of the UCLA VXM Exchange Survey is that, in Phase II, one recipient-donor pair is sent as a blinded sample to the participating laboratory. This pair was originally included in the VXM survey during Phase I and is now used for FXM testing. This setup enables a comparison between FXM and VXM (Figure 2). By October 2023, 58 donor blood samples and 116 well defined HLA reference sera were sent to participating laboratories to peform HLA antibody testing, flow crossmatch (FXM) and VXM since consisting 232 T/B cell FXM pairs and 18 T/B cell VXM pairs. Despite the fact that participating laboratories used different standard operating procedures (SOP) and reagents from different manufacturers, approximately 80% concordance between the VXM predictions and the physical FXM was achieved in the presence of HLA DSA. Significant variability was observed in sera with 1) very high titer antibodies that exit prozone effect; 2) weak-to-moderate DSA, particularly in the presence of multiple weak DSAs; and 3) DSA against lowly expressed antigens. The results were recently summarized and reported in Transplantation (Locke et al., 2023). With the increasing use the VXM, standarization and continuous learning via exchange surveys will provide better understanding and quality controls for VXM to improve accuracy across all centers.

### KIR gene typing

The effects of natural killer (NK) cell alloreactivity on disease relapse and transplant-related mortality following allogeneic stem cell transplantation have been recognized over the past decades (Jennifer Zhang, 2022). Killer immunoglobin-like receptors (KIRs), which recognize HLA class I molecules, are the key receptors in regulating NK cell functions. In 2005, the International KIR Exchange was integrated within the framework of the International Cell Exchange. This was the first program to offer reference DNA samples for KIR genotyping. As background, KIR genes are organized in a complex of loci (∼150–200 kb) in chromosome 19q13.4 (Wilson et al., 2000). The human KIR family consists of 15 KIR genes (*KIR2DL1-4*, *KIR2DL5A*, *KIR2DL5B*, *KIR3DL1-3*, *KIR2DS1-5*, and *KIR3DS1*) and the two pseudogenes (*KIR2DP1*, and *KIR3DP1*). KIR genes are inherited in haplotypes: A haplotype, which consists of nine genes (*3DL3-2DL3-2DP1-2DL1-3DP1-2DL4-3DL1-2DS4-3DL2*) and B haplotypes, which has variable gene content (*2DS1*, *2DS2*, *2DS3*, *2DS5*, *2DL2*, *2DL5*, and *3DS1*) and more activating KIRs. In addition, four framework genes divide the *KIR* haplotypes into centromeric (*KIR3DL3* to *KIR3DP1*) and telomeric (*KIR2DL4* to *KIR3DL2*) regions. Since established in 2004, 248 DNA samples have been shipped with 56 unique haplotypes (Supplementary Table S2). The KIR gene complex is extremely diverse due to allelic polymorphism and gene copy-number variation. Moreover, KIR genes display substantial sequence homology, with a high degree of similarity ranging from 85% to 98% between alleles from any two genes (Roe et al., 2020). This similarity can lead to recombinations and deletions within these genes. These unique features make KIR typing a highly complicated process. Comprehensive analysis of all results using SSP, SSO, real-time PCR and NGS greatly improves the accuracy of the results and the understanding of the KIR haplotypes. For example, *KIR2DS2* has a strong linkage disequilibrium with *KIR2DL2* (Moesta and Parham, 2012). However, exchange sample KIR#231 carries *KIR2DS2* in the CenB haplotype but misses *KIR2DL2*, *KIR2DL5B* and *KIR2DS3* genes as a result of recombination. This recombination also resulted in–KIR2DS2*005 —a hybrid allele sharing the first six exons with *KIR2DS2* and the exons 7 to 9 (cytoplasmic regions) of *KIR2DS3*. KIR2DS2*005 has been reported to present in 1.2% of Caucasoids (Ordonez et al., 2011). On the contrary, in sample #150, *KIR2DL2* is present in the absence of *KIR2DS2*. This haplotype (*KIR2DL1- 2DL2-2DL4-2DL5B-3DL1-3DL2-3DL3-2DS4FULL-2DS5-2DP1-3DP1*_*DEL*_) is exclusively reported in African American population at a frequency of 3.5% (Middleton and Gonzelez, 2010). Among all the samples have been sent, the highest error rate (5 out of 242 samples) was found in *KIR2DS3* with controversial results on the presence or absent of the gene. There are 71 KIR2DS3alleles been documented with 2 null alleles (2DS3*003:01:01N and 2DS3*003:01:02N). *KIR2DS3* can be present on either a Cen-B or Tel-B haplotype. KIR2DS3*003N is identical to KIR2DS3*002 except for a nucleotide change in exon 5 that results in a premature termination. If the primers or probes do not cover exon 5, the KIR2DS3*003N will be mistyped as KIR2DS3*002. The prevalence of KIR2DS3*003N in the Caucasian population has been documented at 0.8% (Luo et al., 2007). 2/246 of samples (#0019 and #0045) did not reach consensus for KIR2DS4 typing due to the inability to distinguish the presence of null alleles. Currently, 41 KIR2DS4 alleles exist and 22 of them are defined based on null alleles. It is reported that KIR2DS4 exists in two versions: one with the full‐length sequence (*001, *016, *017, *018, *020) and the other with a deletion of 22 bp in exon 5 (*003, *004, *006, *007, *008, *009, *010, *012, *013, *014, *015). This deletion leads to a frame shift causing a stop codon in exon 7 which truncates the soluble KIR2DS4 protein. However, no typing errors of *KIR2DS4* have been reported since 2007. Next, *KIR3DL1/S1* gene locus contains reciprocal genes either encoding the inhibitory receptor *KIR3DL1* or the activating receptor *KIR3DS1*. *KIR3DS1* shares >95% homology with *KIR3DL1* in their extracellular domains, yet they have different ligand binding profiles. Unlike *KIR3DL1*, which displays a wide range of polymorphism with a total of 189 alleles, *KIR3DS1* shows a relatively restricted diversity, with 91 alleles having been identified. In the sample KIR#229 *KIR3DL1* typing did not reach consensus with 68% laboratories reported presence while 32% laboratories reported absence of the gene. Since *KIR3DS1* and *KIR3DL1* have identical sequences in exon 3, typing methods purely focusing on exon 3 typing will cause false positive results on the presence of the *KIR3DL1*. Currently, the majority of laboratories use SSP and SSO KIR typing methods. Employing NGS for full-length characterization of KIR genes would undoubtedly enhance typing resolution, but it would also reveal previously undiscovered alleles and haplotypes, advancing the field.

### MICA gene typing

*MHC class I chain-related gene A* (MICA) is a non-conventional MHC-encoded class I molecule located in the HLA complex. Over 500 *MICA* alleles have been reported to date (https://www.ebi.ac.uk/ipd/imgt/hla/about/statistics/). Since its discovery (Bahram et al., 1994; Zhang et al., 2011; Carapito et al., 2022), multiple reports have shown the involvement of MICA in solid organ transplantation (Zou et al., 2007). February 2007, a pilot study for *MICA* genotyping was initiated by sending samples to a select number of laboratories and was expanded to all laboratories a year later. It currently serves as the only proficiency testing program for MICA genotyping, providing an opportunity for laboratories around the world to compare results from different typing methods and to identify new MICA alleles. Since established in 2007, 204 DNA samples have been shipped, including two novel MICA alleles: MICA*018new and MICA*041new. MICA nucleotide variations are mainly located in exons 2, 3, and 4, relating to 3 extracellular domains. MICA exon 5 encodes the transmembrane region (TM) of the MICA protein. It contains the trinucleotide repeat microsatellite polymorphism (GCT)n with eight alleles encoding a variable number of alanine residues: A4, A5, A6, A7, A8, A9, A10, and A5.1. The A5.1 allele contains an extra guanine (G) insertion after 5 GCT repeats, which causes a frameshift leading to a premature stop codon, resulting in a shorter and more easily cleaved protein from the cell surface. 7/204 samples manifested ambiguous results, either because of polymorphism in exons the number of the GCT repeats. For example, the ambiguous result between MICA*007/MICA*026 is due to the number of the GCT repeats, which MICA*007 has 4 GCT repeats compared to 6 GCT repeats in MICA*026. Similarly, ambiguity among MICA*002/*020/*055 is due the number of GCT repeats. MICA*002 has 9 GCT repeats, MICA*020 has 10 GCT repeats, while MICA*055 has 8 GCT repeats. Another common ambiguous involves MICA*009:01 and MICA*049:01. MICA*049:01 differs from MICA*009 only at codon 333 in exon 6 of the cytoplasmic domain by a single nucleotide substitution (ACG- > ATG), which results in an amino acid substitution from threonine to methionine. Continuing exchange programs play a crucial role in collecting essential data that enables the comparison of typing methods and their outcomes across various laboratories, thereby enhancing typing accuracy.

## Future directions

The International Cell Exchange has a long standing history of service and contributions to the field of Histocompatibility and Immunogenetics. With the rapid and continued advancement of technical innovations over the past 50 years, the UCLA International Cell and DNA Exchange Programs endeavor to design, develop and provide up-to-date surveys reviewed by experts in the field to achieve technical and diagnostic relevance.

HLA is the most polymorphic gene complex in the human genome, Wehmeier et al. demonstrated that current SAB panels encompass approximately 98.5% of HLA eplets, yet there is still a lack of representation for HLA alleles within minority populations (Wehmeier et al., 2020). The use of extended SAB antibody detection panels will be important for increasing the precision of HLA antibody detection and improve the accuracy of the VXM, particularly in highly sensitized patients (Zavyalova et al., 2021). Serum samples displaying prozone phenomenon, as well as, sera from patients treated with immune suppressive/immunomodulatory drugs (e.g., rituximab), could be selected as challenges for use in educational surveys. Despite HLA DSA, patients still may lose their grafts due to antibodies directed against non-HLA antigens expressed on the donor endothelium (Zhang and Reed, 2016; Butler et al., 2020)There is a growing need for assays that can identify non-HLA antibodies and their impact on graft injury in context of solid organ transplantation. A future direction for the field will be focusing on the concordance and proficiency in the detection of non-HLA antibodies.

Allorecognition is mediated by T and B lymphocytes, which are responsible for the cellular and humoral mediated immunity, respectively. T and B lymphocytes are activated via the recognition of non-self epitopes by their antigen receptor complexes at their cell surfaces, known as the B cell receptor (BCR) and the T cell receptor (TCR). Recent advances in HLA sequencing technology allows the study of the donor-recipient incompatibility at the molecular level. Still in its infancy, the Predicted Indirectly Recognizable HLA Epitopes (PIRCHE) predicts the T cell epitopes that can be presented by HLA class II molecules to the recipient CD4 T cells (Otten et al., 2013; Lemieux et al., 2022). However, the prediction is currently limited to HLA-DR, and does not include HLA-DQ and DP antigens. Eplets are defined as clusters of polymorphic amino acids situated on the surface of HLA molecules. They serve as functional B cell epitopes, encompassing specific amino acids recognizable by anti-HLA antibodies within the larger amino acid structure comprising an HLA epitope. There are a number of tools that can be used to predict B cell epitopes, including HLAMatchmaker (Duquesnoy, 2001), HLA epitope mismatch algorithm (HLA EMMA) (Kramer et al., 2020) and the three-dimensional electrostatic mismatch score (EMS3D) (Kim et al., 2023). Recent publications suggest that these analyses may provide improved precision in HLA matching and optimal donor selection, and lead to substantial improvements in transplant outcomes and increased graft and patient survival rates (Wiebe et al., 2019; Senev et al., 2020; Mohammadhassanzadeh et al., 2021).

A new direction in the field is Histocompatibility Testing for Swine Leukocyte Antigen (SLA). Histocompatibility testing for xenotransplantation is in its infancy. Methods to detect and define xenoantibodies to swine HLA include flow cytometry crossmatching, complement dependent lymphocytotoxicity and red blood cell agglutination (Ladowski et al., 2021). However, only a few reagents exist to characterize the specificity of the human anti-swine HLA antibodies. Hence, this is a much needed area for clinical research and translation to the clinic to achieve significant advances that will benefit the future of Xenotransplantation.

The future directions of the International UCLA Cell and DNA Exchange Programs will likely involve various aspects of these new ideas.

## Data Availability

The raw data supporting the conclusions of this article will be made available by the authors, without undue reservation.
