# Supplementary figures and images for: Advancing precision in histocompatibility and immunogenetics: a comprehensive review of the UCLA exchange program
Source: Front Genet. 2024 Feb 1;15:1352764. doi: 10.3389/fgene.2024.1352764 (PMC10867271; doi:10.3389/fgene.2024.1352764)

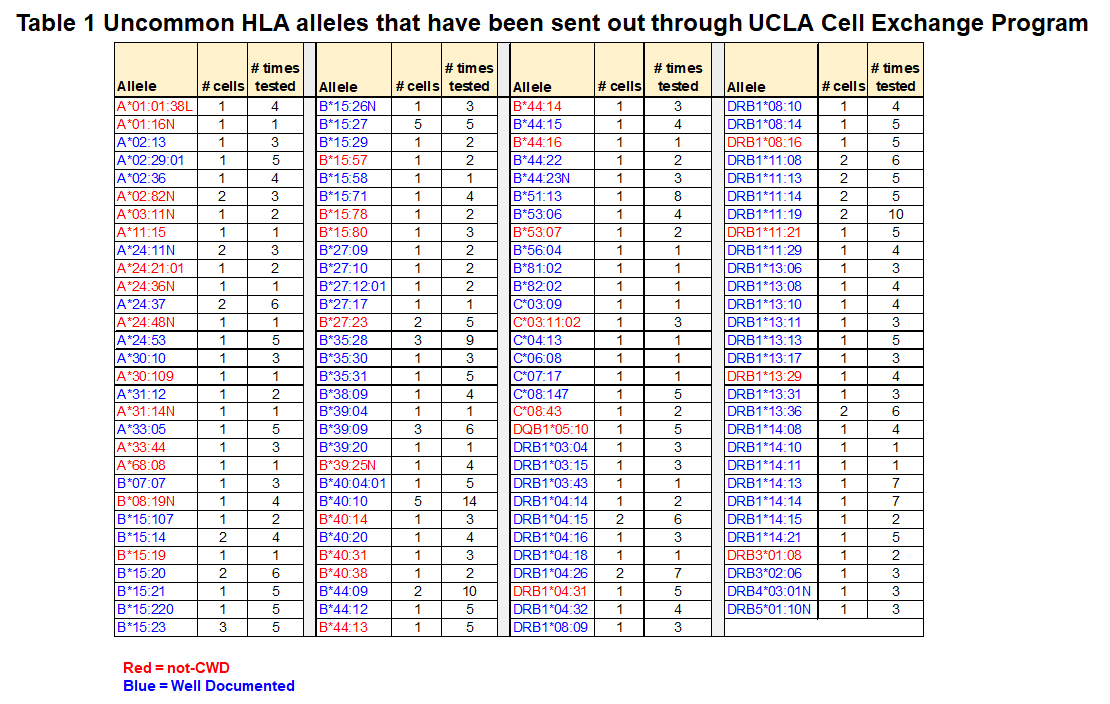

Supplement: Supplementary file 1 [file Image1.JPEG]

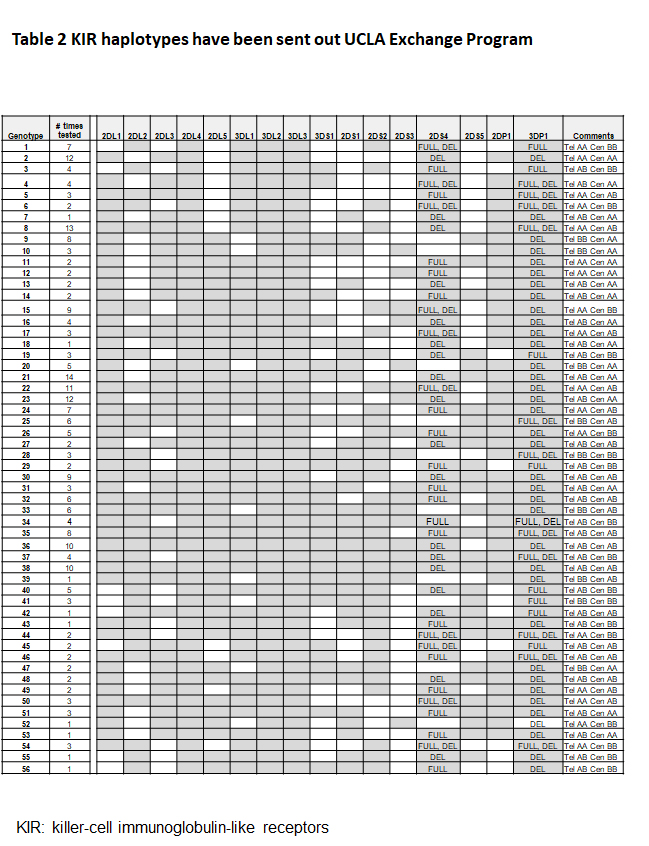

Supplement: Supplementary file 2 [file Image2.JPEG]
